# Fumarate hydratase mutation associated uterine leiomyomas: A case report and literature review

**DOI:** 10.1002/ccr3.8526

**Published:** 2024-04-07

**Authors:** Junyan Zhu, Shanji Li, Zhiguo Zhuang, Hao Chen, Chao Chen, Jie Zhu

**Affiliations:** ^1^ Department of Obstetrics and Gynecology, Ren Ji Hospital Shanghai Jiao Tong University School of Medicine Shanghai China; ^2^ Department of Obstetrics and Gynecology, Shanghai General Hospital Shanghai Jiao Tong University School of Medicine Shanghai China; ^3^ Department of Radiology, Ren Ji Hospital Shanghai Jiao Tong University School of Medicine Shanghai China; ^4^ Department of Pathology, Ren Ji Hospital Shanghai Jiao Tong University School of Medicine Shanghai China

**Keywords:** case report, fumarate hydratase, gene mutation, hereditary leiomyomatosis and renal cell carcinoma, Reed's syndrome, uterine leiomyomas

## Abstract

The patient was found to have multiple uterine myomas at the age of 19, underwent laparoscopic myomectomy at the age of 20, and underwent laparotomic myomectomy again at the age of 23 due to the recurrence of uterine myoma. At the age of 25, the patient reappeared with symptoms and recurrence, and was diagnosed with uterine leiomyomas (ULMs) of FH mutation and high‐grade squamous intraepithelial lesion (HSIL/CIN III) with gland involvement, after complete examination. Fumarate hydratase (FH) mutation screening is important when gynecologists encounter patients with early onset and multiple ULMs, it can give early diagnosis and treatment and fertility guidance. The patient had their uterus removed at the age of 26. FH mutation screening is important when gynecologists encounter patients with early onset and multiple ULMs, it can give early diagnosis and treatment and fertility guidance. It is also helpful for early diagnosis of renal cell carcinoma.

## 1 INTRODUCTION

Uterine leiomyomas (ULMs) are the most common benign tumors in the female reproductive system, and their occurrence often manifests as familial aggregation. Chromosomal abnormalities detected by routine cytogenetic analysis included structural alterations of chromosome 12, translocations between chromosomes 12 and 14, deletions of part of the long arm of chromosome 7, and rearrangement involving the short arm of chromosome 6.^1^ From a genomic perspective, ULMs have at least four major nonoverlapping classes, listed in roughly decreasing order of the main genetic drivers are mediator subcomplex 12 (MED12) point mutation or deletion, high mobility group protein AT‐hook 2 gene (HMGA2) overexpression, fumarate hydratase (FH) inactivation, and COL4A6‐COL4A5 deletion.^2^ Among them, FH encoded by the FH gene, is an enzyme that catalyzes the conversion of fumarate to malate in the Krebs cycle. Germline mutations in FH predispose women to early onset and multiple ULMs. Here we report a case with a novel FH mutation carriers suffer from ULMs.

## 2 CASE PRESENTATION

### 2.1 Chief complaints

A 26‐year‐old patient presented to our outpatient clinic and requested a hysterectomy.

### 2.2 History of present illness

The patient's symptoms again started 6 months prior, including menorrhagia, lower abdominal pain, and frequent urination.

### 2.3 History of past illness

Seven years ago, the patient was diagnosed with uterine myoma at the age of 19. One year later, laparoscopic myomectomy was performed in a local hospital due to the rapid growth of uterine myoma. The postoperative pathology was uterine leiomyoma, and treatment with gonadotropin‐releasing hormone analog (GnRH‐a) agonist for half a year. Three years ago, the patient underwent laparotomic myomectomy in Japan due to abnormal uterine bleeding and recurrence of uterine myoma. The postoperative pathology was still uterine leiomyoma. Two years ago, uterine myoma were found to recur again in the follow‐up ultrasound examination, and received GnRH‐a treatment again for half a year.

Seven months ago, follow‐up ultrasound examination showed multiple uterine myomas, the largest size was 4.8 × 3.9 cm, HPV16(+), TCT was LSIL, and HSIL could not be excluded. A cervical biopsy was performed and the pathology report was high‐grade squamous intraepithelial lesion (HSIL/CIN III) with gland involvement. Five months ago, the patient underwent cold‐knife conization in a hospital in Beijing. Postoperative pathology was cervical tissue with chronic inflammation, HSIL at 4 and 5 o'clock, glandular accumulation at 4 o'clock, small foci of HSIL at 8 o'clock, HSIL at 6 and 5 o'clock, LSIL at 7 o'clock, and the incision is clean.

### 2.4 Family history

The patient's mother, grandmother, and aunt underwent hysterectomy for multiple uterine myoma. No kidney and skin tumors were found in the patient's immediate family.

### 2.5 Physical examination

Pelvic examination showed an irregularly enlarged uterus, similar to the size of 3 months of pregnancy, with a hard texture. No skin nodules were found on physical examination.

### 2.6 Laboratory examinations

Blood analysis revealed mild anemia, with a hemoglobin level of 99g/L. Serum tumor markers (CA125, CA199, CEA, AFP, SCC) were normal.

### 2.7 Imaging examinations

**FIGURE 1:**
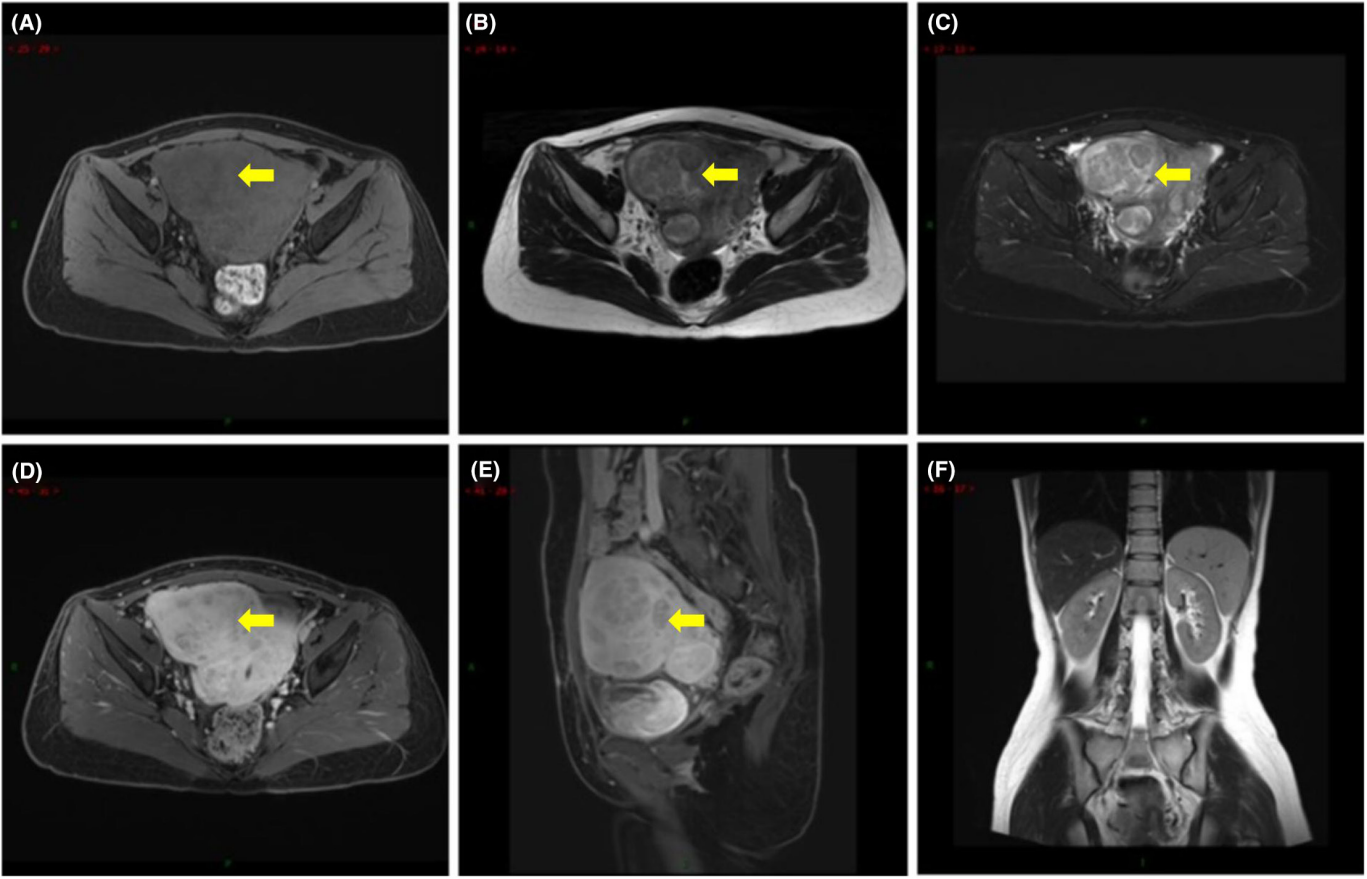
Magnetic resonance imaging (MRI) before hysterectomy. (A) Axial fat‐saturated precontrast T1‐weighted image showing intermediate signal intensity foci. (B) Axial T2‐weighted image showing intermediate and hypointense foci. (C) Axial fat‐saturated T2‐weighted image showing intermediate and hypointense foci. (D) Axial fat‐saturated postcontrast T1‐weighted image showing inhomogenous enhancement of the foci and lower signal intensity of the foci compared to the myometrium. (E) Sagittal fat‐saturated postcontrast T1‐weighted image showing inhomogenous enhancement of the foci and lower signal intensity of the foci compared to the myometrium. (F) Coronal T2‐weighted image showing normal kidney.

Ultrasound showed that the volume of the uterus was significantly increased. The size of the uterus was approximately 8.1 × 9.7 × 9.9 cm, and the myometrium were multiple hypoechoic masses of variable sizes, the largest size is 7.1 × 7.6 × 7.7 cm. Pelvic magnetic resonance imaging (MRI) showed enlarged uterus and multiple nodules in the uterus, the largest one was located in the anterior wall of the uterus, the size of the lesion was about 8 × 7.6 × 6.5 cm. It was isointense on T1‐weighted image, slightly hypointense on T2‐weighted image, and enhanced to varying degrees after enhancement (Figure 1). In addition, MRI showed no obvious abnormalities in both kidneys at present.

### 2.8 Next‐generation sequencing (NGS)

Genetic testing of postoperative paraffin specimens and peripheral blood confirmed FH mutation (mutations of undetermined significance). Germline and somatic FH mutation c.928A>G (p.N310D) was identified, somatic FH mutation c.667A > G (p.K223E) was identified.

## 3 FINAL DIAGNOSIS

The final diagnosis was ULMs of FH mutation and HSIL/CIN III with gland involvement.

## 4 TREATMENT

**FIGURE 2:**
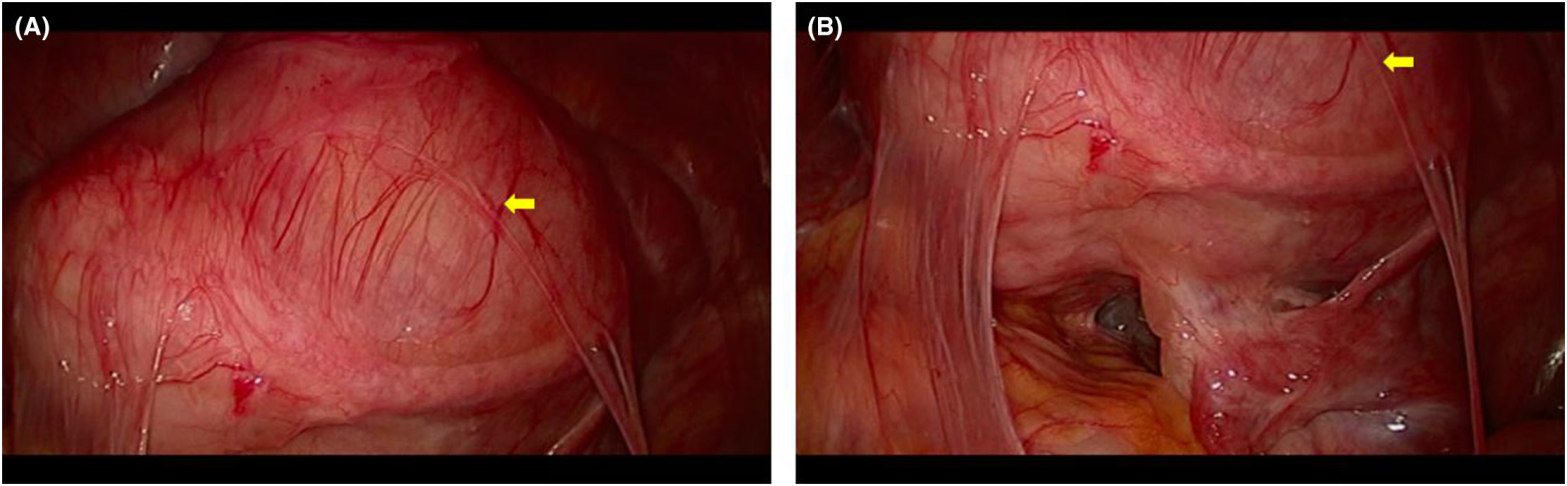
Laparoscopic findings. (A) The uterus was enlarged, such as the size of 3 months of pregnancy. (B) The appearance of the double accessories was normal, and all of them adhered to the peritoneum, and extensive adhesion of the bowel.

**FIGURE 3:**
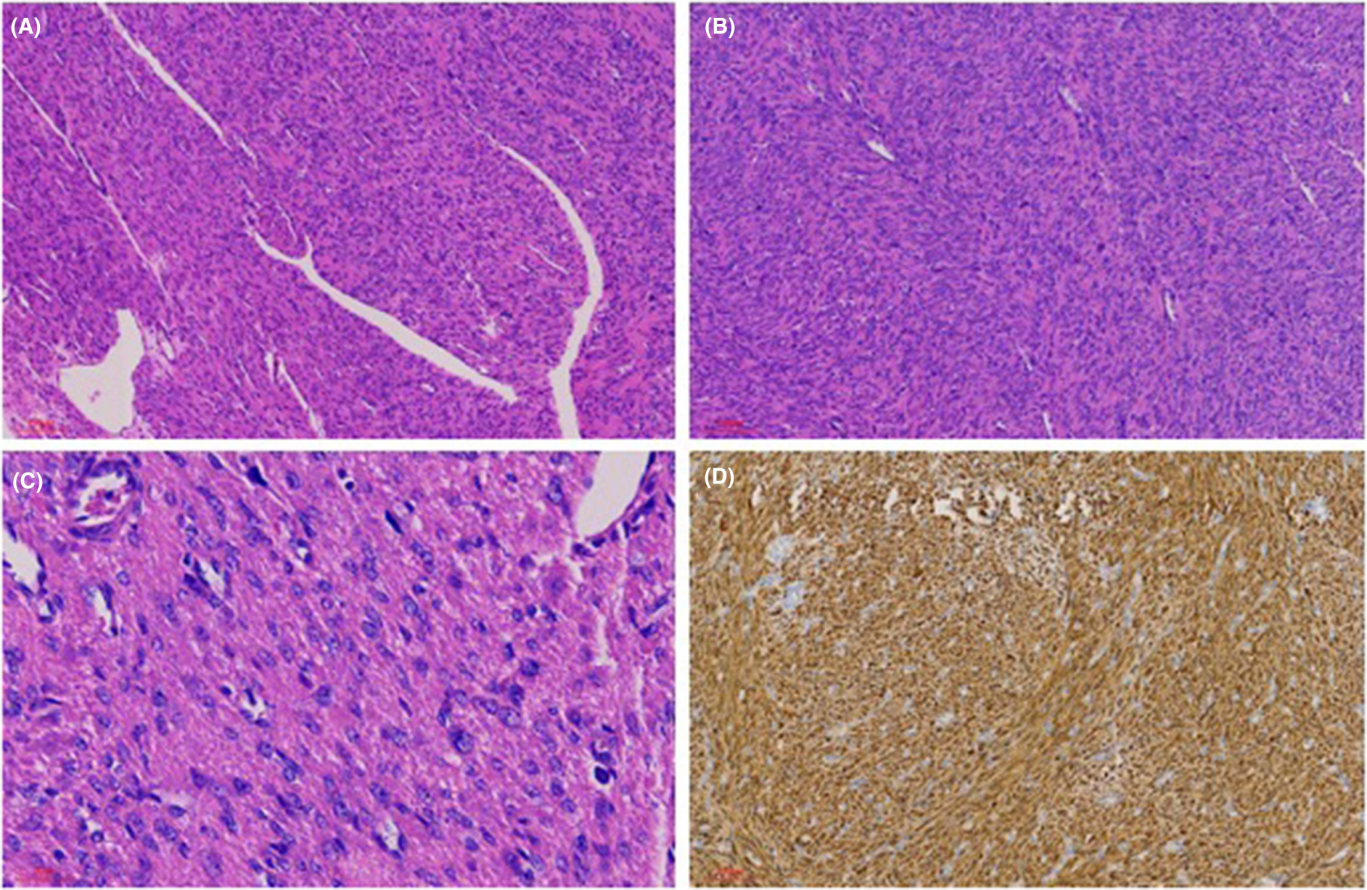
Pathological features of FH mutation ULMs. It displayed well circumscribed, fascicular tumors with increased cellularity, nuclear atypia, and occasional mitoses. (A) Staghorn vessels at 40× original magnification. (B) Fibrillary cytoplasm at 100× original magnification. (C) Eosinophilic globules at 400× original magnification. (D) IHC by 2SC staining positive at 100× original magnification.

The patient underwent laparoscopic total hysterectomy and bilateral salpingectomy after admission in February 2022. Intraoperative exploration revealed extensive adhesion of the bowel to the anterior peritoneum, the uterus was enlarged, such as the size of 3 months of pregnancy, the appearance of the double accessories was normal, and all of them adhered to the lateral peritoneum (Figure 2). The operation went well and the bleeding was about 100 mL. Postoperative paraffin pathology consistent with pathological features of FH‐mutated ULMs (Figure 3).

## 5 OUTCOME AND FOLLOW‐UP

The patient was followed up to 3 months after the operation and recovered well, and the abdominal wound and vaginal stump wound healed well.

## 6 DISCUSSION

Uterine smooth muscle tumors (USMTs) include a wide spectrum of benign, atypical/uncertain malignant potential, and fully malignant tumor types. Each tumor type has variants with distinct cytohistologic and molecular profiles. Recent advances in next‐generation sequencing (NGS) identify driver gene mutations and genomic alterations in many USMT types, allowing further tumor classification and more accurate diagnosis. ULMs account for the most common USMT type in reproductive‐age women.^3^ Many ULMs and their variants are driven by MED12 and HMGA2 mutations/alterations, while a small fraction are caused by alterations in FH and COL4A5‐COL4A6.^4^, ^5^, ^6^

FH gene is associated with different types of ULMs, among which hereditary leiomyomatosis and renal cell carcinoma (HLRCC) is the only well‐defined syndrome, also known as Reed's syndrome. HLRCC is a rare autosomal dominant syndrome caused by germline mutations in the FH gene with increased risks of cutaneous leiomyomas (CLMs) and uterine leiomyomas (ULMs) and renal cell carcinoma (RCC).^7^, ^8^ Previously, the incidence may have been underestimated due to insufficient awareness of the disease. In 2016, HLRCC‐associated RCC is recognized as a separate category in the World Health Organization (WHO) classification of renal tumors, it has been widely recognized.^9^ Although CLMs frequently precede ULMs, cutaneous nodules may be inconspicuous, and therefore symptomatic ULMs may be the primary reason to seek medical care.^8^ Diagnosis that begins with a thorough personal and family history, and a thorough skin examination, and if applicable, a gynecologic examination should be performed.^8^ Although genetic testing is currently considered the criterion standard to diagnose, immunohistochemistry (IHC) could be used as a screening method in tumors with features suggestive of FH alterations,^10^, ^11^ newer IHC markers may have a role in provide rapid and cost effective testing,^8^, ^12^ an expert pathologic assessment will facilitate the clinical identification of FH mutation cases.^13^ FH mutation associated ULMs have been shown to demonstrate distinctive pathological features delineating them from ULMs in the general population. They are well circumscribed, fascicular tumors with increased cellularity, nuclear atypia, and occasional mitoses.^8^, ^14^ Fibrillary cytoplasm, eosinophilic globules, and staghorn vessels may also be found.^8^, ^15^ A characteristic finding is tumor nuclei with orangiophilic (i.e., eosinophilic) inclusion‐like nucleoli with a perinuclear halo. This finding is the hallmark of FH mutation ULMs, and should prompt additional workup if clinical features or history suggests HLRCC.^8^, ^16^ when patient's medical history and/or histopathologic tumor characteristics indicate potential FH‐deficiency, the tumor's FH status is determined by 2SC staining. When aberrant staining is observed, the patient can be directed to genetic counseling and mutation screening.^12^

Mutations in the FH gene are not uniquely associated with HLRCC syndrome. The great majority of HLRCC patients' ULMs are caused by FH inactivation, but incidental tumors driven by somatic MED12 mutations also occur.^17^ FH inactivation can also occur in sporadic ULMs.^10^ No cutaneous or renal cell tumors were reported when it was referred to genetic analyses in a family with closely related women with ULMs, and a novel germline FH mutation was detected.^18^ A recent study showed that a relatively high rate of FH germline mutation in FH‐negative ULMs from patients aged up to 30 years.^19^ Some cases describe in young patient who was originally diagnosed with atypical leiomyoma (ALM) from a myomectomy specimen and experienced recurrence many years later, suggestive of FH mutations.^20^ WHO (2014) classification defines ALM as leiomyoma with bizarre nuclei (LBN). Another study showed that FH gene mutations were a common finding only in LBN, but not in ULMs and leiomyosarcoma (LMS).^11^ There are also data demonstrate that HLRCC‐related morphological features, abnormal FH/2SC staining, and somatic FH mutations/deletions can be seen in a subset of sporadic tumors in LBN.^21^ Inactivation of FH can occur in nonsyndromic ULMs but is rare in other tumors.^22^

The patient had a family history of ULMs, but neither the patient nor her family members had CLMs and RCC. At the age of 19, the patient presented with menstrual changes as the first symptom and was found to have early onset and multiple ULMs. After the onset of the disease, twice myomectomy and drug treatment were performed in a short period of time, and then it recurred again, until genetic testing identifies the FH germline mutation. What is lacking is that gynecologists failed to think of genetic testing to confirm the diagnosis at an early stage, resulting in repeated treatment and recurrence in a short time, failure to do reproductive guidance.

## 7 CONCLUSIONS

Whether HLRCC syndrome associated ULMs or sporadic ULMs, FH gene deficiency may play an important role in its pathogenesis. FH mutation screening is important when gynecologists encounter patients with early onset and multiple ULMs, it can give early diagnosis and fertility guidance. It is also helpful for early diagnosis of RCC. It is believed that with the continuous progress of basic and clinical research on FH gene, it can provide guidance for the clinical diagnosis of patients with ULMs in the near future, and providing a new direction for the treatment of ULMs.

## AUTHOR CONTRIBUTIONS

**Junyan Zhu:** Writing – original draft. **Shanji Li:** Writing – original draft. **Zhiguo Zhuang:** Formal analysis. **Hao Chen:** Data curation. **Chao Chen:** Conceptualization. **Jie Zhu:** Writing – review and editing.

## FUNDING INFORMATION

Supported by Shanghai Municipal Health Commission and Shanghai Muricipal Administrator of Traditional Chinese Medicine, No. ZHYY‐ZXYJHZX‐201904.

## CONFLICT OF INTEREST STATEMENT

None of the authors have any conflict of interest to report.

## CONSENT

Written informed consent was obtained from the patient to publish this report in accordance with the journal's patient consent policy.

## Data Availability

All the data for used are available from the patients archive of Ren Ji Hospital and after coordination with corresponding author.
